# Bioinformatics analysis of lncRNA‑associated ceRNA network in melanoma

**DOI:** 10.7150/jca.51851

**Published:** 2021-03-15

**Authors:** Yi Ding, Min Li, Tuersong Tayier, MeiLin Zhang, Long Chen, ShuMei Feng

**Affiliations:** 1 Department of Histology and Embryology, School of Basic Medical Sciences, Xinjiang Medical University, Urumqi, Xinjiang, China.; 2 Department of Pharmacology, Pharmacy College, Xinjiang Medical University, Urumqi, China.; 3 Xinjiang Urumqi City Center Blood Station, Urumqi, China.

**Keywords:** Bioinformatics analysis, ceRNA, differentially expressed gene, melanoma, survival analysis.

## Abstract

Melanoma is an extremely malignant tumor with early metastasis and high mortality. Little is known about the process of by which melanoma occurs, as its mechanism is very complex and only limited data are available on its long non-coding RNA (lncRNA)-associated competing endogenous RNAs (ceRNAs). The purpose of this study was to screen out potential prognostic molecules and identify a ceRNA network related to the occurrence of melanoma. We screened 169 differentially expressed mRNAs (DEmRNAs) from E-MTAB-1862 and GSE3189; gene ontology (GO) enrichment analysis showed that these genes were closely related to the development of skin. In the protein-protein interaction network, we screened out a total of 19 hub genes. Furthermore, we predicted the microRNAs (miRNAs) that regulate hub genes using the miRWalk database and then intersected these with GSE35579, resulting in nine DEmiRNAs. We also predicted the lncRNAs that regulate the miRNAs using the LncBasev.2 database. According to the ceRNA hypothesis, and based on the intersection of the DElncRNAs with merged GTEx and TCGA data, we obtained 20 DElncRNAs. A total of four DEmRNAs, nine DEmiRNAs, and 20 DElncRNAs were included in the ceRNA network. Based on Cox stepwise regression and survival analysis, we identified five biomarkers, ZSCAN16-AS1, LINC00520, XIST, DTL, and let-7a-5p, and obtained risk scores. The results showed that most of the differentially expressed genes were related to epithelial-mesenchymal transition (EMT) in melanoma. Finally, we obtained a LINC00520/let-7a-5p/DTL molecular regulatory network. These results suggest that ceRNA networks have an important role in evaluating the prognosis of patients with melanoma and provide a new experimental basis for exploring the EMT process in the development of melanoma.

## Introduction

Melanoma is the most frequently occurring malignancy in the elderly and has high mortality rates 1. In recent decades, the number of patients with melanoma has increased with the aging of the population 2. The development of melanoma is caused by the progression of a benign nevus, sometimes accompanied by sun exposure, physical stimulation, genetic variation, and other factors 3. Nevi are benign products of normal melanocytes in the skin. In general, nevi will not develop into melanoma 4. However, constant rubbing or physical stimulation of the skin in the affected area increases the probability of malignant transformation. At present, treatments for melanoma include surgery, radiotherapy, chemotherapy, and immunotherapy, but their efficacy is not optimal 5-7. The transformation of a normal nevocytic nevus into melanoma is regulated by a complex molecular network. Therefore, it is of great importance to clarify the molecular mechanism of melanoma formation and find new molecular therapeutic targets. This study aimed to unravel the pathogenesis, prognostic factors, and potential molecular therapeutic targets of melanoma. First, we used multiple public databases to screen out differentially expressed genes (DEGs), mRNAs (DEmRNAs), microRNAs (DEmiRNAs), and long non-coding RNAs (DElncRNAs). Then, we constructed a competing endogenous RNA (ceRNA) network based on gene enrichment, target gene prediction, survival analysis, and multivariate Cox regression analysis. Finally, five predictive genes, ZSCAN16-AS1, LINC00520, XIST, DTL, and let-7a-5p were identified; these genes are closely related to the occurrence of melanoma. This study provides new molecular therapeutic targets and lays the foundation for a better understanding of the mechanism of melanoma formation.

## Materials and Methods

### Data preparation

We downloaded two expression profile datasets, E-MTAB-1862 and GSE3189, from the European Bioinformatics Institute ArrayExpress database (http://www.ebi.ac.uk/arrayexpress) and the Gene Expression Omnibus (GEO, http://www.ncbi.nlm.nih.gov/geo/) database, respectively. The E-MTAB-1862 dataset consisted of 11 nevus samples and 21 melanoma samples 8, based on the A-AFFY-44-Affymetrix GeneChip Human Genome U133 Plus 2.0 array. The GSE3189 dataset, submitted by Talantov et al., included 18 nevi samples and 45 melanoma samples 9 and was based on the GPL96 [HG-U133A] Affymetrix Human Genome U133A array. In addition, miRNA sequencing data for melanoma was downloaded as GSE35579; this dataset incorporates 11 benign nevi and 20 primary melanoma samples, sequenced on the GPL15183 CRUK/Melton lab-Human melanoma-71-v2-microRNA expression profiling platform. Finally, we obtained lncRNA sequencing data from the merged GTEx and The Cancer Genome Atlas (TCGA) databases (https://www.cancergenome.nih.gov/). The GTEx melanoma data was downloaded from UCSC Xena (http://xena.ucsc.edu/) 10. As of June 20, 2020, a total of 813 normal samples and 471 melanoma samples were merged.

### Identification of DEGs in melanoma

After downloading the mRNA expression profiles of E-MTAB-1862, we used the EdgeR package in R version 4.0.0 to identify DEmRNAs^11^. Then, we used the ggplot2 R package to draw a heat map and volcano plot of the DEmRNAs. Next, we filtered the DEmRNAs from GSE3189 using the GEO2R software 12. To find overlapping DEmRNAs, we used the Venn software online to obtain the intersection of the two datasets. Moreover, we used GEO2R to screen DEmiRNAs from GSE35579 between nevi tissue and melanoma tissue. Finally, after merging the GTEx and TCGA data using R, we filtered out DElncRNAs with the EdgeR package, and generated the corresponding heat map and volcano map using ggplot2. The DEmRNAs and DEmiRNAs were then filtered using adjusted p<0.05 and |log fold change (FC)|>1, and the DElncRNAs with thresholds of |log FC|>1, p <0.05, and false discovery rate (FDR) < 0.05.

### Functional enrichment analysis

GO annotation analysis and Kyoto Encyclopedia of Genes and Genomes (KEGG) pathway analysis were performed using the STRING (Search Tool from the Retrieval of Interacting Genes; https://string-db.org/) database (version 11.0) 13. STRING can be used to predict protein-protein interactions (PPI) directly or indirectly. (p < 0.05 and FDR<0.05).

### PPI analysis

We used the STRING database to visually evaluate DEmRNAs with a confidence score >0.4. Furthermore, we used the Cytoscape software (version 3.7.2) to visualize the PPI 14. We used the Molecular Complex Detection (MCODE) application in Cytoscape to screen hub genes of the PPI network with degree >8 (degree of cutoff = 2, node score cutoff = 0.2, k-core = 2, maximal depth = 100).

### Construction of ceRNA regulation network in melanoma

We used the miRWalk and miRDB databases to predict the miRNAs that regulate DEmRNAs with mini p-value >0.9 15. To find overlapping DEmiRNAs, we used Venn software online to identify the intersection between the predicted DEmiRNAs and those from the GSE35579 dataset. Furthermore, we predicted lncRNAs that regulated the DEmiRNAs using the LncBasev.2 database with threshold >0.9 16. Then, we identified the intersection of the DElncRNAs obtained after merging the GTEx and TCGA databases with the predicted lncRNAs. Finally, based on the ceRNA hypothesis, we constructed a ceRNA network and visualized the results using Cytoscape.

### Survival analysis of DEGs

We used the GEPIA database (http://gepia.cancer-pku.cn/index.html) to analyze the expression of particular lncRNAs and mRNAs in melanoma 17. Then, we carried out a survival analysis of specific lncRNAs, miRNAs, and mRNAs using the oncolnchttp website (http://www.oncolnc.org/) 18 with log-rank p <0.05.

### Multivariate Cox regression model analysis

Only DEGs identified as meaningful in the survival analysis were considered for further analysis. Based on the clinical information of patients with survival status and duration in the TCGA database. First of all, we rule out those patients who have no survival status. Furthermore, we used the survival R package to perform multivariate Cox regression analysis using the six meaningful DEGs (LINC00520, ZNF561-AS1, XIST, MMP9, DTL, and let-7a-5p). We also performed a stepwise regression analysis of these six variables and obtained risk scores. To evaluate the prognostic value of the final gene combination in patients with melanoma, patients were divided into high- and low-risk groups based on the median risk score. Moreover, survival analysis and receiver operating characteristic (ROC) curve analysis of the final gene combination were carried out using the survival and survivalROC R packages, respectively. Finally, we calculated the area under the curve (AUC). The heat map of the six DEGs was constructed using ggplot2 (p<0.05). To better predict the prognosis of patients, we used melanoma patients with gender, stage, and TNM stages in the TCGA database as inclusion criteria. At the same time, we used a survival package combined with clinical information of patients and the risk model constructed in this experiment for univariate and multivariate COX analyses and obtained the related forest plots. Furthermore, the ROC curve related to clinical information was drawn to evaluate the prognostic value of the model. P-value < 0.05.

## Results

### Identification of DEGs in melanoma

**Figure 1:**
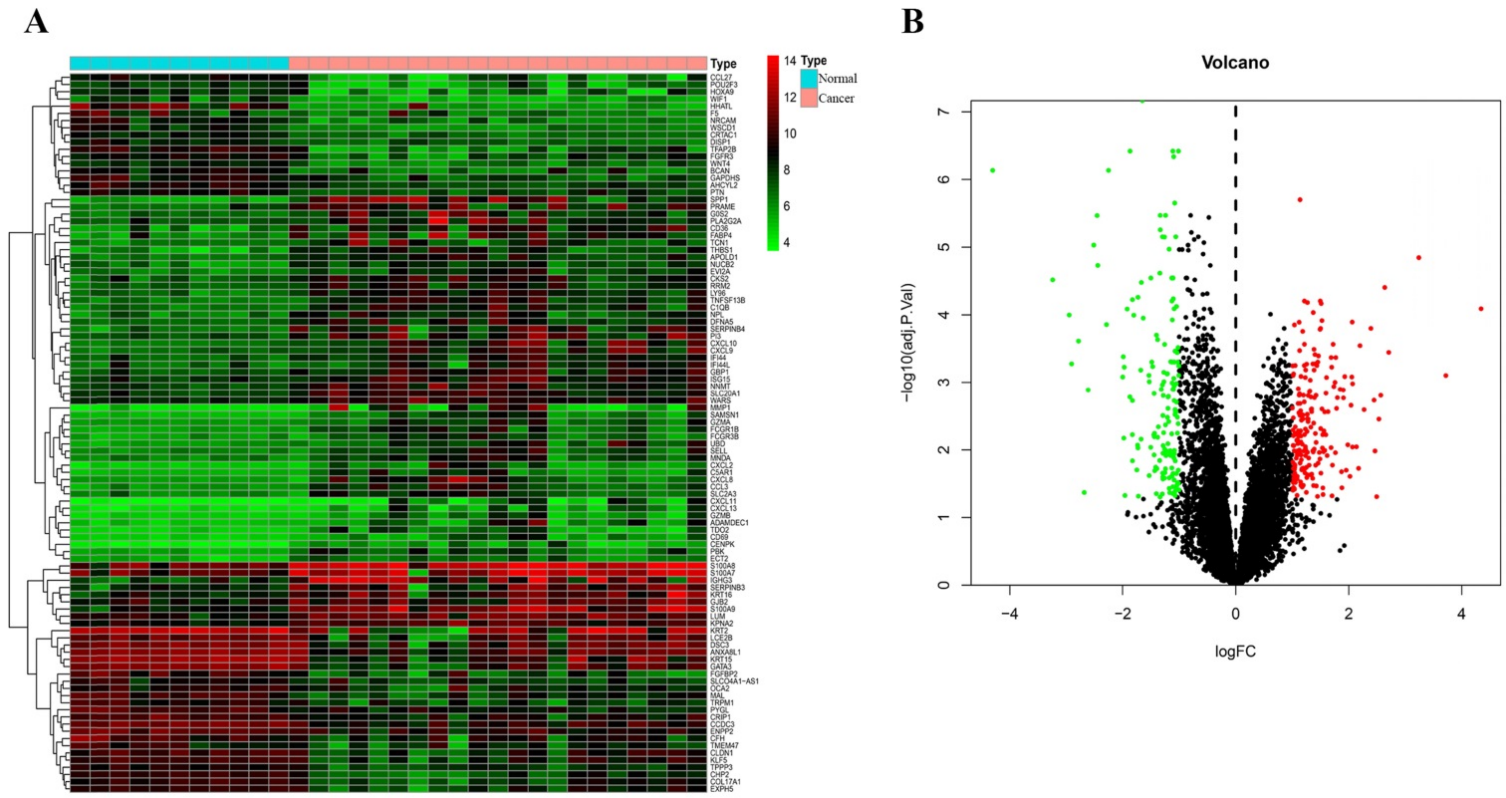
** Distribution of DEmRNAs in melanoma.** (A) Heat map of the first 100 DEmRNAs. (B) Volcano plot of DEmRNAs. Red indicates upregulation, green indicates downregulation, and black indicates normal expression in the volcano plot. |log FC|>1, adjusted p<0.05.

**Figure 2:**
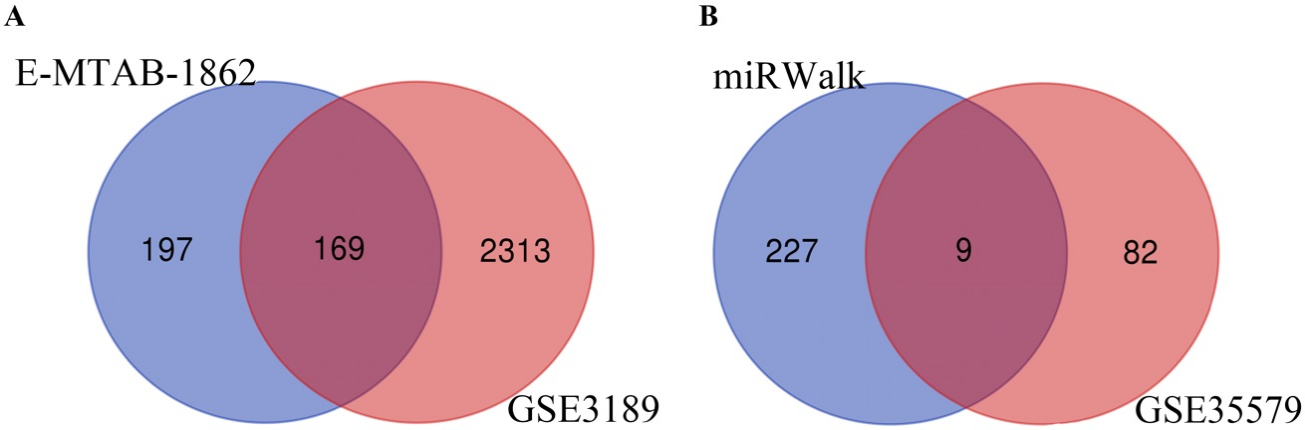
** Venn diagram.** (A) 169 DEmRNAs; (B) nine DEmiRNAs.

**Figure 3:**
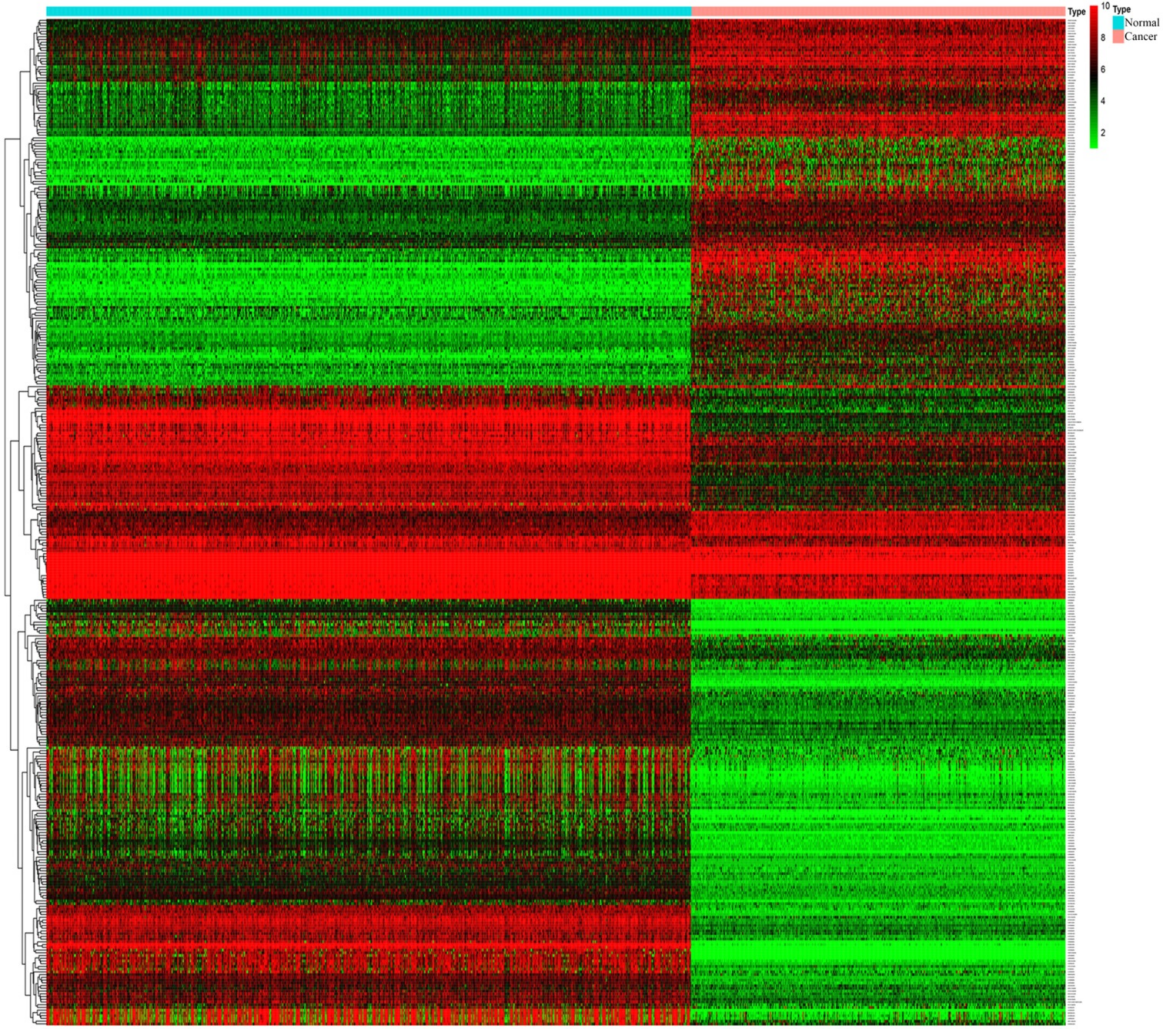
** Heat map of DElncRNAs.** |log FC|>1, p<0.05, and FDR< 0.05.

Through analysis of gene expression profiles in E-MTAB-1862, we screened a total of 367 DEGs, of which 210 were upregulated and 156 were downregulated in melanoma. Then, we used a volcano plot and heatmap to map the top 100 DEmRNAs **(Figure 1)**. We also screened 3090 DEmRNAs from the GSE3189 dataset using GEO2R, of which 1215 were upregulated and 1875 were downregulated in melanoma tissues. Finally, we took the intersection of the two datasets using the Venn software online and found that a total of 169 DEmRNAs appeared in both datasets, of which 74 were upregulated and 95 were downregulated in melanoma samples **(Figure 2a)**. In addition, we analyzed the miRNA sequencing data from the GSE35579 dataset; 91 DEmiRNAs were screened, of which 31 were upregulated and 60 were downregulated. We identified nine miRNAs in the intersection of GSE35579 with the DEmiRNAs predicted from the miRWalk database **(Figure 2b)**. We also screened DElncRNAs by combining the melanoma GTEx and TCGA data, and obtained 684 upregulated and 1160 downregulated DElncRNAs in melanoma. The ggplot2 R package was used to plot the heat map of DElncRNAs **(Figure 3)**.

### GO term and KEGG pathway analysis

**Figure 4:**
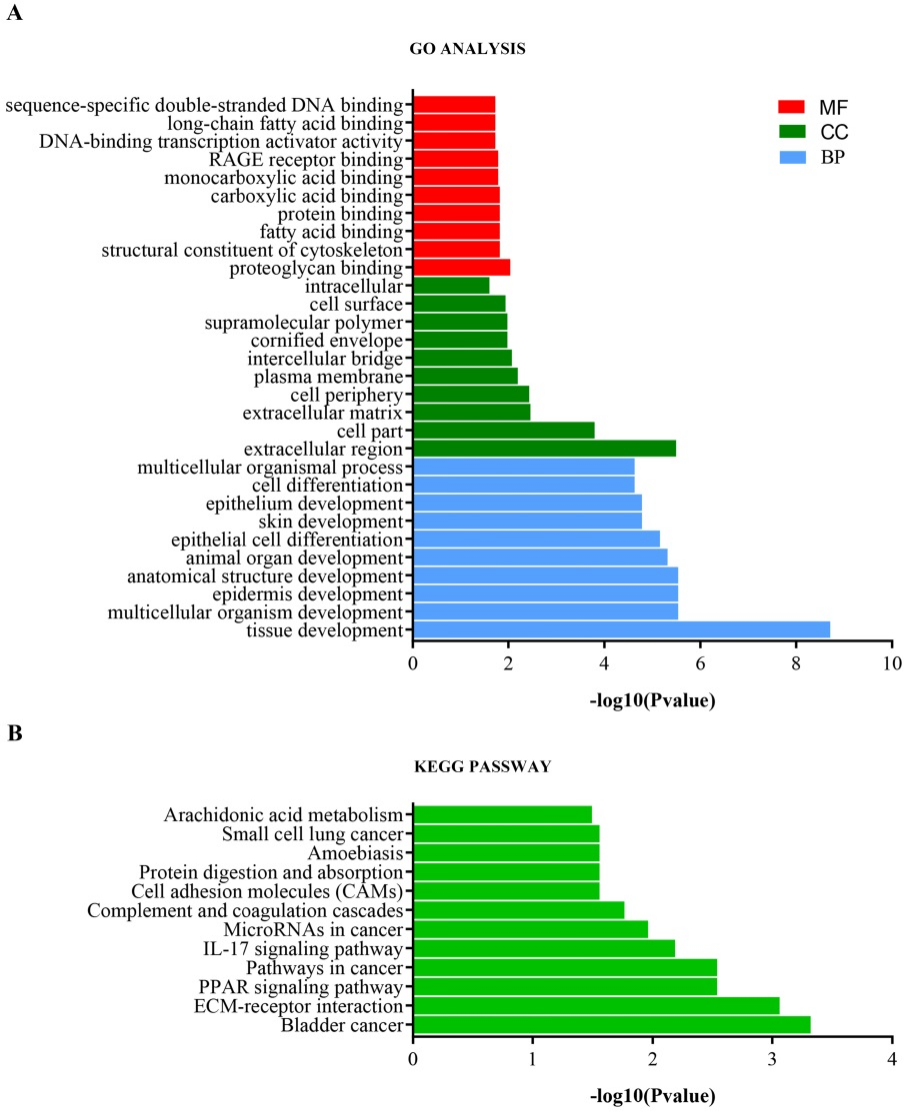
** GO and KEGG pathway enrichment analysis of DEmRNAs.** (A) GO enrichment analysis. BP, biological process; CC, cellular component; MF, molecular function. (B) KEGG pathway enrichment analysis.

Analysis of the top 10 GO enrichment results **(Table 1, Figure 4a)** showed that the DEmRNAs were mainly involved in tissue development, epidermis development, skin development, and cell differentiation with respect to biological processes. Regarding cellular components, the DEmRNAs were mainly enriched in the extracellular matrix, intercellular bridge, cornified envelope, and cell surface. Regarding molecular functions, the DEmRNAs were significantly involved in proteoglycan binding, structural constituent of the cytoskeleton, carboxylic acid binding, and DNA-binding transcription activator activity. Furthermore, KEGG pathway analysis showed that the DEmRNAs were involved in bladder cancer, ECM-receptor interaction, PPAR signaling pathway, pathways in cancer, IL-17 signaling pathway, and microRNAs in cancer **(Table 1, Figure 4b)**.

### PPI analysis

**Figure 5:**
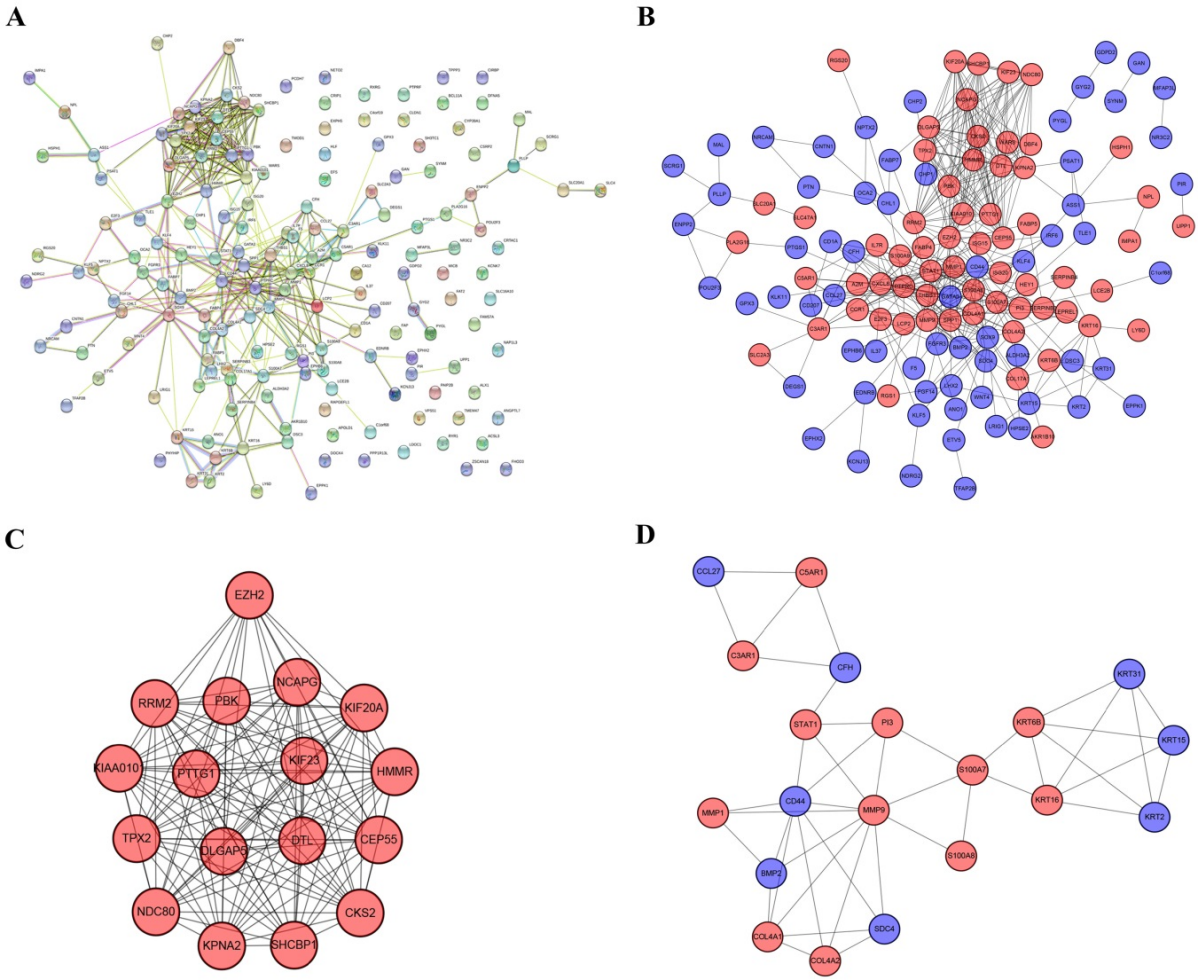
** PPI network, most significant DEmRNA module, and interaction network of hub genes.** (A) PPI network of 169 DEmRNAs constructed in STRING. (B) PPI network of DEmRNAs constructed with Cytoscape. (C-D) Two modules obtained from the PPI network using MCODE Cytoscape plug-in. Red nodes represent upregulated genes, blue nodes represent downregulated genes.

The PPI network contained 169 nodes and 407 edges, including 74 upregulated genes and 95 downregulated genes **(Figure 5a)**. We used Cytoscape to visualize the PPI network. Furthermore, we used MCODE to identify two modules comprising 37 genes. Then, taking degree >8 as the screening index **(Figure 5b)**, we selected 19 genes, of which the CD44 gene had low expression and the other genes had high expression in melanoma.

### Constructing the ceRNA regulation network in melanoma

**Figure 6:**
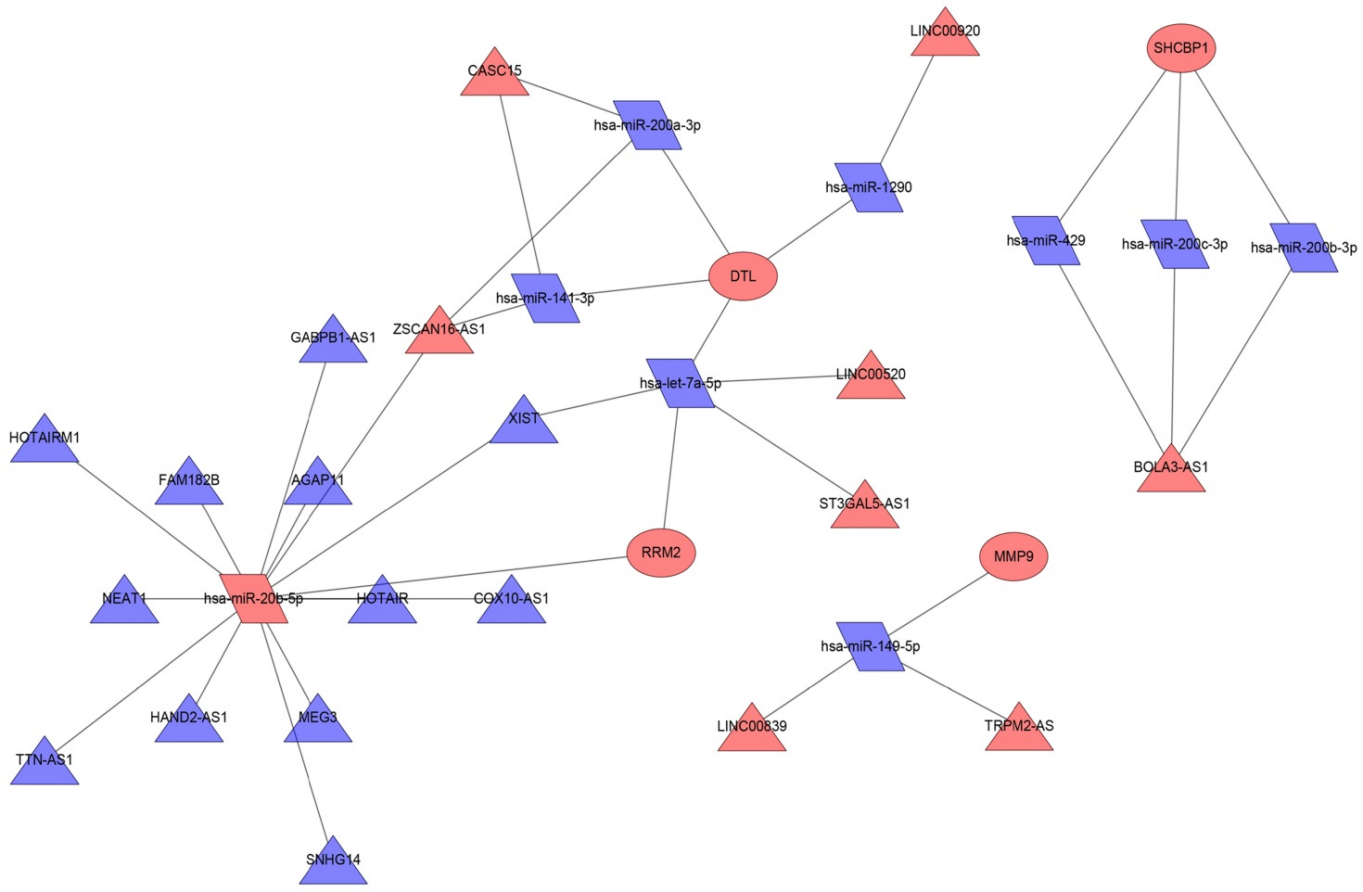
** ceRNA network in melanoma.** Red nodes indicate increased expression, blue nodes indicate decreased expression; rectangles represent mRNAs, rhomboids represent miRNAs, and triangles represent lncRNAs.

We used the miRWalk database to predict the miRNAs regulating 19 mRNAs **(Table 2)**; these miRNAs were also found in the miRDB database. Then, we took the intersection of the miRNAs in the predicted miRNA database and GSE35579 dataset, resulting in nine miRNAs **(Table 3)**. Furthermore, we used the LncBasev.2 database to predict the lncRNAs regulating the miRNAs (threshold >0.8), and used the Venn software online to intersect the different lncRNAs from the merged GTEx and TCGA data with the predicted lncRNAs. According to the ceRNA hypothesis, there is a positive regulatory relationship between lncRNAs and mRNAs, and a negative regulatory relationship between miRNAs and mRNAs. Finally, we obtained 20 lncRNAs with significant expression differences in melanoma **(Table 4)**. We constructed the ceRNA network using Cytoscape **(Figure 6)**.

### Survival analysis of DEGs

**Figure 7:**
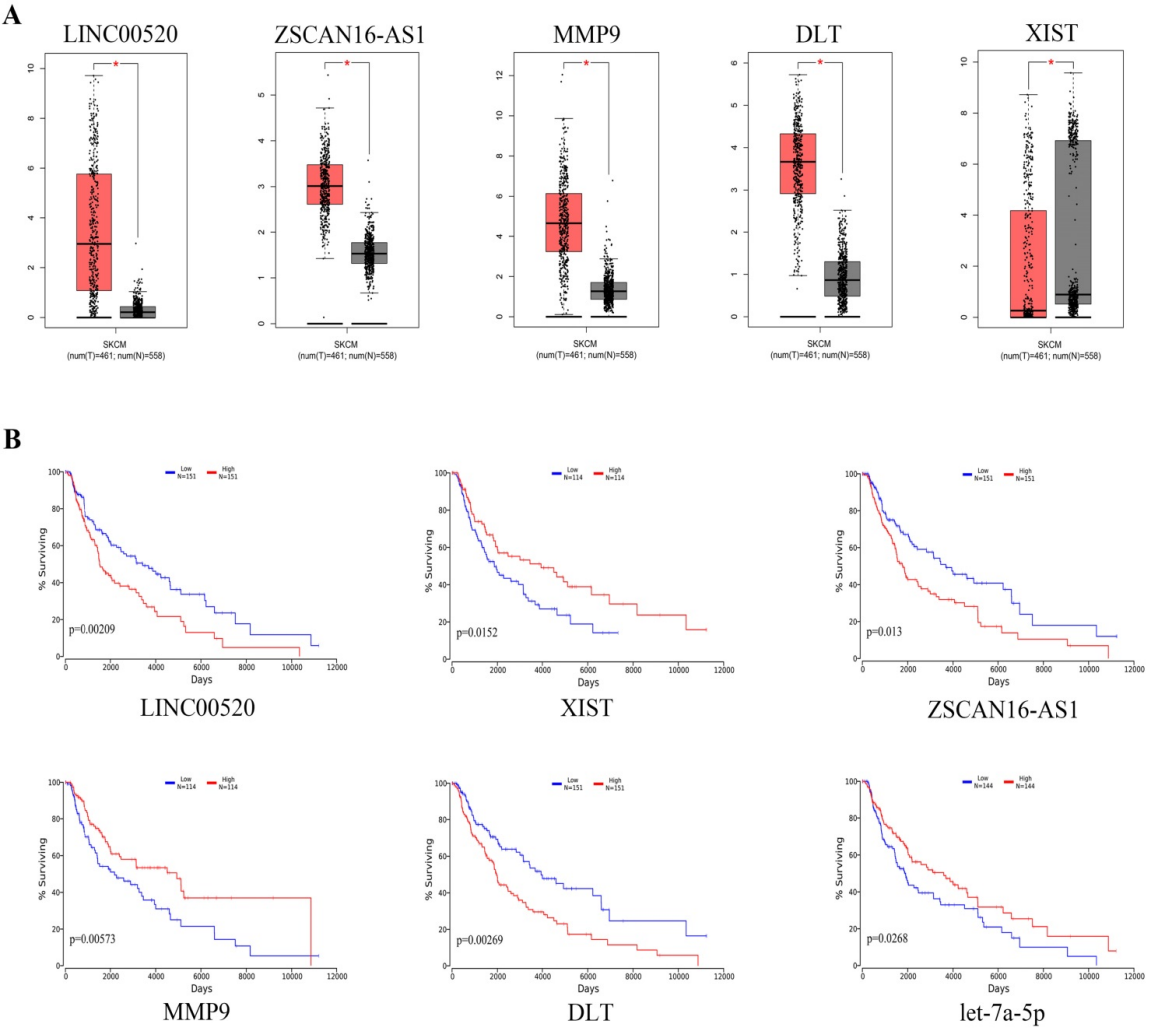
** Expression levels and survival analysis of specific genes in melanoma compared with normal melanocytes.** (A) Expression levels of specific genes in melanoma according to GEPIA (*p<0.05). (B) Survival analysis for specific genes by oncolnchttp (log-rank p<0.05).

We used GEPIA to analyze the expression of specific DEGs **(Figure 7a)**. The results showed that compared with normal nevus tissues, LINC00520, ZSCAN16-AS1, MMP9, and DTL had higher expression in melanoma, whereas XIST had lower expression. Survival analysis using the oncolnchttp website showed that LINC00520, ZSCAN16-AS1, XIST, MMP9, DTL, and let-7a-5p were significantly correlated with overall survival (OS) **(Figure 7b)**. Kaplan-Meier survival analysis showed that the survival time of patients with high expression of LINC00520, ZSCAN16-AS1, MMP9, and DTL was significantly shortened, whereas that of patients with high expression of XIST and let-7a-5p was significantly prolonged (log-rank p<0.05).

### Multivariate Cox regression analysis

**Figure 8:**
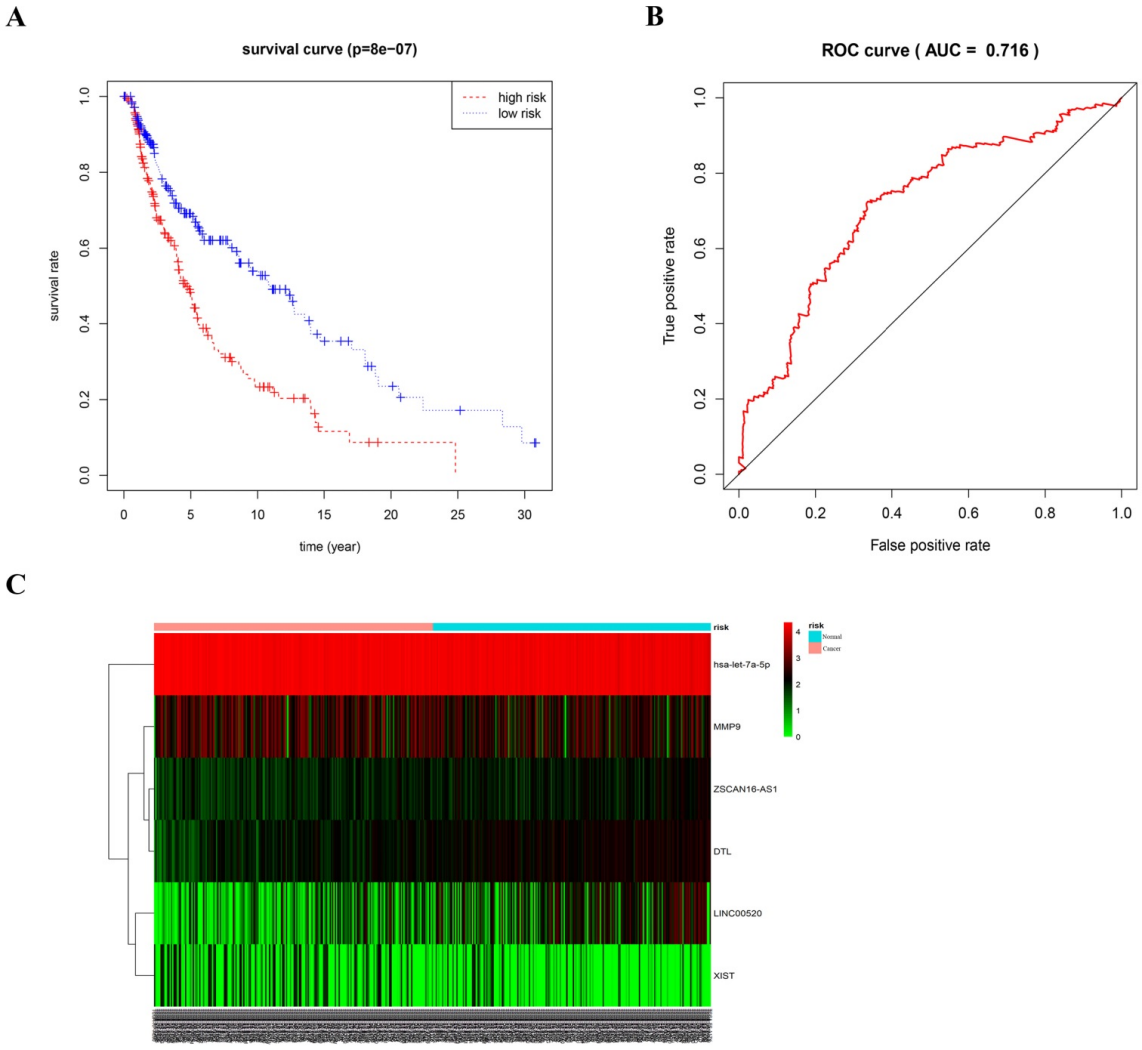
** ROC curve and heat map of five gene combinations.** (A) OS curves for five gene combinations in melanoma. (B) ROC curve and AUC. (C) Heat map of the five gene combinations.

As different marker combinations appeared very promising as prognostic indicators, we further evaluated the prognostic value of six gene combinations in patients with melanoma. Using stepwise regression analysis of six variables, we obtained five variables by Cox regression. Risk scores were calculated as follows, risk score=0.68192*ZSCAN16-AS1+0.14792*LINC00520+0.74931*DTL-3.07155*let-7a-5p-0.12864*XIST. To further evaluate the prognostic value of the five gene combinations in patients with melanoma, patients were divided into high- and low-risk groups according to the median of the Cox regression model. Kaplan-Meier survival analysis showed that the survival time of patients in the high-risk group was significantly shorter than that in the low-risk group (p<0.05). ROC curve analysis showed that a combination of four genes had high accuracy and specificity in evaluating the prognosis of patients with melanoma, with AUC=0.716. The expression of five genes in the high and low-risk groups was visualized using a heat map **(Figure 8)**. Also, univariate COX analysis of the risk model and clinical information showed that both stage and TNM stages could predict the prognosis of patients with melanoma (Supplemental Figure S1A). The forest plot results of multivariate COX analysis showed that T and N could be used as independent prognostic factors (Supplemental Figure S1B). However, the ROC results showed that the AUC of the risk model, T and N was 0.705, 0.679, and 0.667, respectively (Supplemental Figure S1C). Therefore, compared with TNM staging, the risk model we constructed is more accurate in predicting patient survival.

## Discussion

Epithelial-mesenchymal transformation (EMT) is the process by which epithelial cells transform into mesenchymal cells. Under normal physiological conditions, EMT is related to human embryonic development, wound healing, and tissue regeneration 19. In the pathological state, EMT is associated with cancer progression and fibrosis, and is significantly related to the occurrence and development of cancer. During the development of cancer, because of gene mutations, adhesion molecules and connections between cells are reduced, the extracellular matrix is also reduced, and the cytoskeleton is reconstructed 20. Therefore, cancer cells can migrate and invade, and eventually spread to other organs. Moreover, cancer cells further promote metastasis by changing the tumor microenvironment and secreting enzymes that degrade the extracellular matrix. EMT-treated cells also gain the ability to resist apoptosis. In this study, we screened 169 DEmRNAs, 91 DEmiRNAs, and 1844 DElncRNAs. The results of the GO analysis showed that the function of DEmRNAs was mainly related to epidermis development, proteoglycan binding, and structural constituents of the cytoskeleton; these factors are closely related to the occurrence of melanoma. Notably, the top 10 biological process terms were all related to skin development. With respect to molecular functions, most of the DEmRNAs were related to protein binding and structural constituents of the cytoskeleton. Therefore, we speculate that the formation of melanoma may be closely related to changes in the cytoskeleton structure of normal nevus cells.

In addition, the KEGG pathway analysis showed that the DEmRNAs were mainly enriched in bladder cancer, ECM-receptor interactions, the PPAR signaling pathway, and the IL-17 signaling pathway. Surprisingly, we found that most of the DEmRNAs were related to ECM-receptor interaction, which is closely related to cell adhesion and migration. Generally, EMT is related to the metastatic and invasive ability of cancer cells. During EMT 21, the integrity of normal epithelial cells is lost, the adhesion and connections between cells are reduced, and the cytoskeleton is reconstructed, which leads to greater movement, migration, and invasion of cancer cells. Furthermore, we found that most of the genes were enriched in the bladder cancer pathway, suggesting a significant relationship between bladder cancer and melanoma.

After using the PPI network to cluster DEmRNAs, we found that among the 19 hub genes, only CD44 showed low expression in melanoma. CD44 can interact with moesin protein, which has been shown to promote the migration and infiltration of breast cancer cells by remodeling the cytoskeleton 22, thereby further promoting the metastatic ability of cancer cells 23, 24. CD44 is a cell surface glycoprotein that is highly expressed in some cancer stem cells and is closely related to cell motility and cancer metastasis. In normal nevocytic nevus, CD44 shows strong staining. In melanoma, the expression of CD44 decreases gradually with increasing invasiveness 25. DTL regulates the cell cycle and maintains the stability of the genome. DTL is highly expressed in invasive hepatocellular carcinoma and is closely related to tumor grade and prognosis 26. Chen et al. found that knocking down the expression of DTL in liver cancer inhibited the growth and invasion of liver cancer cells, accelerated the apoptosis of cancer cells, and inhibited tumorigenesis 27. The same effects were found in gastric cancer, breast cancer, and cervical cancer 28. Through bioinformatics analysis, Zhou et al. identified DTL as a hub gene in hepatocellular carcinoma cells and found that it was highly expressed 26. Through survival analysis, a close relationship was identified between DTL and the prognosis of patients with liver cancer. In addition, previous studies have reported that DTL is highly expressed in melanoma compared with benign melanocytic nevi and significantly correlated with OS 29. However, the effects of DTL on the migration and invasion of melanoma have not been reported.

MMP9 is a matrix metalloproteinase that promotes the migration and invasion of cancer cells by degrading the extracellular matrix 30. MMP9 has been reported to play an important part in the EMT process of cancer cells. Its overexpression can increase the invasive ability of hepatocellular carcinoma cells and promote metastasis 31, leading to higher TNM staging and poor prognosis. High expression of MMP9 is often found in breast cancer 32; in malignant breast cancer tissues, MMP9 shows strong positive expression. Overexpression of MMP9 can enhance the colony formation and migration ability of breast cancer cells, whereas inhibition of MMP9 leads to a significant decrease in invasive ability. Furthermore, survival analysis showed that MMP9 has a significant relationship with prognosis in breast cancer patients and could thus represent a prognostic biomarker for breast cancer 33. In addition, MMP9 can promote angiogenesis; Li et al. showed that the angiogenic ability of melanoma cells could be inhibited by knocking down the expression of MMP9 34. There is a well known positive correlation between angiogenesis and invasion of cancer cells. Bakos et al. showed that the expression of MMP9 was low in normal melanocytes 35. Therefore, we infer that MMP9 plays an important part in the transformation of normal melanocytes into melanoma.

In this study, we also constructed a lncRNA-related ceRNA network to identify related miRNAs in melanoma. The functions of lncRNAs have been implicated in a variety of cancers. LINC00520 has been widely reported in breast cancer, colorectal cancer, skin squamous cell carcinoma, melanoma, and other cancer cells, and shows increased expression in breast cancer tissue compared with normal breast tissue. Henry et al. showed that the migration ability of breast cancer cells could be blocked by knocking down the expression of LINC00520 36. LINC00520 can also promote the proliferation of cutaneous squamous cell carcinoma, as well as enhancing its migration and invasiveness via upregulating the expression of MMP9 37. Wen et al. found that LINC00520 had a similar function in melanoma. Compared with normal melanocytes, LINC00520 was highly expressed in melanoma 38, and overexpression of LINC00520 could promote the proliferation, migration, and invasion of melanoma cells. In addition, it was significantly associated with a shorter OS in patients with melanoma. The role of XIST in cancer has also been widely reported. Tian et al. showed that XIST was highly expressed in melanoma cells 39. Overexpression of XIST can promote the proliferation, migration, and invasion of melanoma cells. Moreover, XIST is associated with TNM stage and lymphatic metastasis in patients with melanoma. We showed here for the first time that lncRNA ZSCAN16-AS1 was highly expressed in patients with melanoma and significantly correlated with poor OS. To the best of our knowledge, no role of ZSCAN16-AS1 has previously been reported in any disease. Let-7a-5p is a gene that has been studied extensively and is been found in a variety of cancer cells. In the current study, it was found to regulate downstream target genes. Functional experiments with let-7a-5p showed that its overexpression could inhibit the proliferation, migration, and invasion of lung cancer cells 40, as well as promoting autophagy and apoptosis. Li et al. found that let-7a-5p inhibited the proliferation of lung cancer cells by inhibiting the G1/S phase process 41. Moreover, let-7a-5p was associated with shorter OS in patients with lung cancer. The miRNA sequencing analysis by Babapoor et al. showed that the expression of let-7a-5p in invasive melanoma was lower than that in normal melanocytes 42.

Different from previous studies, our study focused more on the relationship between the ceRNA network and melanoma epithelial-mesenchymal transformation (EMT). Also, the data sources of this study were more abundant. For example, mRNA and miRNA data come from GEO and ArrayExpress databases, and lncRNA data comes from TCGA and GTEx databases. Moreover, we not only constructed a melanoma ceRNA network but also carried out multivariate COX analysis of the key molecules in the ceRNA network. Finally, a risk model was constructed. To evaluate the clinical value of this risk model, through multivariate COX analysis, combined with the clinical data of melanoma patients in the TCGA database, we found that this risk model can predict the survival of melanoma patients more accurately than traditional TNM staging. To independently verify our risk model, we query the melanoma data from online databases such as the GEO database. Unfortunately, most data sets only sequence mRNA or miRNA and lack relevant lncRNA data, and some melanoma data lack patient survival data. Therefore, this study has not been able to independently verify this risk model. However, we will still strive to collect tissue samples and clinical information of melanoma patients in the future, and further, verify our risk model through gene chip sequencing data.

In the current study, we found that DTL, MMP9, LINC00520, and ZSCAN16-AS1 were highly expressed in melanoma compared with normal melanocytes, whereas XIST and let-7a-5p showed the opposite trend. The results of the survival analysis showed that these six genes were closely related to the prognosis of patients with melanoma. Furthermore, the results of our multivariate Cox analysis suggested that the combination of DTL, LINC00520, ZSCAN16-AS1, XIST, and let-7a-5p could identify high-risk melanoma patients. According to the ceRNA network, both LINC00520 and XIST can bind to let-7a-5p, and let-7a-5p can interact with its target gene DTL. However, previous studies showed that XIST was highly expressed in melanoma, contrary to our results. The role of XIST in melanoma has not been fully elucidated; we will investigate this in future research. Finally, we constructed a ceRNA network for LIINC00520/let-7a-5p/DTL.

In summary, we constructed a ceRNA network using miRNA, lncRNA, and mRNA expression profiles in melanoma and normal nevus. This will provide a basis for subsequent studies of the regulation mechanism of melanoma. In addition, we identified a combination of five genes that could represent a new signature for the diagnosis and prognostic analysis of patients with melanoma. In the future, we will further expand the collection of clinical data based on these data, and conduct a more detailed analysis of the pathogenesis of melanoma to clarify the role of these genes in the ceRNA network.

## Supplementary Material

Supplementary figures and tables.

## Figures and Tables

**Table 1:** GO and KEGG pathway enrichment analysis of DEmRNAs.

Term ID	Term description	Observed counts	FDR
Biological processes			
GO:0009888	Tissue development	46	1.95E-09
GO:0007275	Multicellular organism development	76	2.91E-06
GO:0008544	Epidermis development	19	2.91E-06
GO:0048856	Anatomical structure development	80	2.91E-06
GO:0048513	Animal organ development	55	4.84E-06
GO:0030855	Epithelial cell differentiation	23	6.93E-06
GO:0043588	Skin development	17	1.66E-05
GO:0060429	Epithelium development	29	1.66E-05
GO:0030154	Cell differentiation	59	2.35E-05
GO:0032501	Multicellular organismal process	90	2.35E-05
Cell component			
GO:0005576	Extracellular region	50	3.20E-06
GO:0044464	Cell part	161	1.60E-04
GO:0031012	Extracellular matrix	11	3.50E-03
GO:0071944	Cell periphery	69	3.70E-03
GO:0005886	Plasma membrane	67	6.40E-03
GO:0045171	Intercellular bridge	5	8.50E-03
GO:0001533	Cornified envelope	5	1.05E-02
GO:0099081	Supramolecular polymer	19	1.05E-02
GO:0009986	Cell surface	16	1.15E-02
GO:0005622	Intracellular	141	2.49E-02
Molecular function			
GO:0043394	Proteoglycan binding	5	9.20E-03
GO:0005200	Structural constituent of cytoskeleton	7	1.51E-02
GO:0005504	Fatty acid binding	4	1.51E-02
GO:0005515	Protein binding	82	1.51E-02
GO:0031406	Carboxylic acid binding	9	1.51E-02
GO:0033293	Monocarboxylic acid binding	5	1.64E-02
GO:0050786	RAGE receptor binding	3	1.64E-02
GO:0001228	DNA-binding transcription activator activity	12	1.87E-02
GO:0036041	Long-chain fatty acid binding	3	1.87E-02
GO:1990837	Sequence-specific double-stranded DNA binding	17	1.87E-02
KEGG			
hsa05219	Bladder cancer	6	4.80E-04
hsa04512	ECM-receptor interaction	7	8.70E-04
hsa03320	PPAR signaling pathway	6	2.90E-03
hsa05200	Pathways in cancer	15	2.90E-03
hsa04657	IL-17 signaling pathway	6	6.50E-03
hsa05206	Micrornas in cancer	7	1.09E-02
hsa04610	Complement and coagulation cascades	5	1.72E-02
hsa04514	Cell adhesion molecules (cams)	6	2.77E-02
hsa04974	Protein digestion and absorption	5	2.77E-02
hsa05146	Amoebiasis	5	2.77E-02
hsa05222	Small cell lung cancer	5	2.77E-02
hsa00590	Arachidonic acid metabolism	4	3.20E-02

**Table 2:** Expression of 19 mRNAs in melanoma.

EXPRESSION	GENES	logFC		adj.P.Val	
	**mRNA**	**E-MTAB-1862**	**GSE3189**	**E-MTAB-1862**	**GSE3189**
**Upregulated**	DTL	1.30	1.37	1.37E-03	6.89E-08
	SHCBP1	1.22	1.30	2.73E-04	8.49E-07
	KPNA2	1.51	1.13	6.81E-05	5.37E-12
	DLGAP5	1.23	1.60	4.92E-03	1.06E-08
	KIAA0101	1.12	1.52	1.29E-04	3.14E-09
	CEP55	1.28	2.02	1.57E-03	1.39E-11
	HMMR	1.05	1.23	4.56E-03	4.80E-06
	NDC80	1.40	2.15	5.32E-04	8.88E-07
	RRM2	1.57	1.58	2.75E-03	1.02E-11
	EZH2	1.37	1.01	9.25E-05	1.33E-04
	KIF23	1.27	3.85	3.46E-03	8.07E-14
	KIF20A	1.16	1.98	1.40E-02	2.15E-10
	PBK	1.76	1.20	1.00E-03	2.09E-08
	PTTG1	1.02	1.67	3.36E-03	4.74E-15
	TPX2	1.07	1.52	5.92E-03	2.17E-12
	NCAPG	1.15	2.45	5.24E-03	1.87E-10
	CKS2	1.93	1.88	8.21E-04	6.15E-12
	MMP9	1.49	1.30	1.36E-02	1.10E-03
**Downregulated**	CD44	-1.13	-0.83	2.85E-05	1.23E-03

**Table 3:** Information about nine miRNAs in the ceRNA network.

miRNA	logFC	adj.P.Val
hsa-miR-149-5p	-2.34	4.04E-06
hsa-miR-429	-1.65	4.38E-02
hsa-miR-1290	-1.73	5.03E-03
hsa-miR-141-3p	-2.07	3.18E-03
hsa-miR-200c-3p	-1.47	2.39E-05
hsa-miR-200b-3p	-3.43	2.87E-06
hsa-miR-200a-3p	-2.69	1.21E-05
hsa-miR-20b-5p	2.34	4.62E-04
hsa-let-7a-5p	-1.00	1.57E-02

**Table 4:** The list of 20 DElncRNAs in the ceRNA network.

lncRNA	logFC	pValue	FDR
LINC00839	1.74	5.23E-87	1.16E-86
TRPM2-AS	1.05	1.11E-72	2.14E-72
BOLA3-AS1	1.69	2.62E-159	1.43E-158
LINC00920	2.79	3.17E-184	3.2E-183
CASC15	1.29	5.5E-128	1.85E-127
ZSCAN16-AS1	1.61	4.18E-159	2.26E-158
HOTAIR	-1.24	3.45E-124	1.1E-123
AGAP11	-1.24	6.98E-181	6.38E-180
FAM182B	-1.08	1.6E-101	4E-101
XIST	-1.73	4.98E-56	8.23E-56
NEAT1	-4.94	1.57E-194	3.9E-193
HAND2-AS1	-1.44	3.14E-185	3.45E-184
MEG3	-5.04	2.2E-190	3.33E-189
GABPB1-AS1	-1.19	7.81E-139	3.06E-138
HOTAIRM1	-1.02	1.28E-87	2.86E-87
TTN-AS1	-2.50	8.34E-196	3.82E-194
SNHG14	-1.52	1.57E-171	1.12E-170
COX10-AS1	-1.64	3.33E-192	6.33E-191
LINC00520	2.13	5.8E-113	1.63E-112
ST3GAL5-AS1	1.25	4.24E-172	3.09E-171

## References

[1] Wei CY, Zhu MX, Lu NH (2019). Bioinformatics-based analysis reveals elevated MFSD12 as a key promoter of cell proliferation and a potential therapeutic target in melanoma. Oncogene.

[2] Cheng PF (2018). Medical bioinformatics in melanoma. Curr Opin Oncol.

[3] Sun C, Wang L, Huang S (2014). Reversible and adaptive resistance to BRAF(V600E) inhibition in melanoma. Nature.

[4] Damsky WE, Bosenberg M (2017). Melanocytic nevi and melanoma: unraveling a complex relationship. Oncogene.

[5] Lim SY, Lee JH, Diefenbach RJ (2018). Liquid biomarkers in melanoma: detection and discovery. Mol Cancer.

[6] D'Arcangelo D, Giampietri C, Muscio M (2018). WIPI1, BAG1, and PEX3 Autophagy-Related Genes Are Relevant Melanoma Markers. Oxid Med Cell Longev.

[7] Roszik J, Markovits E, Dobosz P (2019). TNFSF4 (OX40L) expression and survival in locally advanced and metastatic melanoma. Cancer Immunol Immunother.

[8] Eriksson J, Le Joncour V, Nummela P (2016). Gene expression analyses of primary melanomas reveal CTHRC1 as an important player in melanoma progression. Oncotarget.

[9] Talantov D, Mazumder A, Yu JX (2005). Novel genes associated with malignant melanoma but not benign melanocytic lesions. Clin Cancer Res.

[10] Goldman MJ, Craft B, Hastie M (2020). Visualizing and interpreting cancer genomics data via the Xena platform. Nat Biotechnol.

[11] Debroas G, Hoeffel G, Reynders A (2018). [Neuroimmune interactions in the skin: a link between pain and immunity]. Med Sci (Paris).

[12] Barrett T, Wilhite SE, Ledoux P (2013). NCBI GEO: archive for functional genomics data sets-update. Nucleic Acids Res.

[13] Szklarczyk D, Gable AL, Lyon D (2019). STRING v11: protein-protein association networks with increased coverage, supporting functional discovery in genome-wide experimental datasets. Nucleic Acids Res.

[14] Shannon P, Markiel A, Ozier O (2003). Cytoscape: a software environment for integrated models of biomolecular interaction networks. Genome Res.

[15] Sticht C, De La Torre C, Parveen A (2018). miRWalk: An online resource for prediction of microRNA binding sites. PLoS One.

[16] Paraskevopoulou MD, Vlachos IS, Karagkouni D (2016). DIANA-LncBase v2: indexing microRNA targets on non-coding transcripts. Nucleic Acids Research.

[17] Tang Z, Li C, Kang B (2017). GEPIA: a web server for cancer and normal gene expression profiling and interactive analyses. Nucleic Acids Research.

[18] Anaya J (2016). OncoLnc: linking TCGA survival data to mRNAs, miRNAs, and lncRNAs. PeerJ Computer Science.

[19] Nieto MA, Huang RY, Jackson RA (2016). Emt: 2016. Cell.

[20] Singh M, Yelle N, Venugopal C (2018). EMT: Mechanisms and therapeutic implications. Pharmacol Ther.

[21] Lamouille S, Xu J, Derynck R (2014). Molecular mechanisms of epithelial-mesenchymal transition. Nat Rev Mol Cell Biol.

[22] Wang CC, Liau JY, Lu YS (2012). Differential expression of moesin in breast cancers and its implication in epithelial-mesenchymal transition. Histopathology.

[23] Ichikawa T, Masumoto J, Kaneko M (1998). Moesin and CD44 expression in cutaneous melanocytic tumours. Br J Dermatol.

[24] Martin TA, Harrison G, Mansel RE (2003). The role of the CD44/ezrin complex in cancer metastasis. Critical Reviews in Oncology/Hematology.

[25] Harwood CA, Green MA, Cook MG (1996). CD44 expression in melanocytic lesions: a marker of malignant progression?. Br J Dermatol.

[26] Zhou Z, Li Y, Hao H (2019). Screening Hub Genes as Prognostic Biomarkers of Hepatocellular Carcinoma by Bioinformatics Analysis. Cell Transplant.

[27] Chen YC, Chen IS, Huang GJ (2018). Targeting DTL induces cell cycle arrest and senescence and suppresses cell growth and colony formation through TPX2 inhibition in human hepatocellular carcinoma cells. Onco Targets Ther.

[28] van Dam PA, Rolfo C, Ruiz R (2018). Potential new biomarkers for squamous carcinoma of the uterine cervix. ESMO Open.

[29] Yang L, Dai J, Ma M (2019). Identification of a functional polymorphism within the 3'-untranslated region of denticleless E3 ubiquitin protein ligase homolog associated with survival in acral melanoma. Eur J Cancer.

[30] Yang F, Yu N, Wang H (2018). Downregulated expression of hepatoma-derived growth factor inhibits migration and invasion of prostate cancer cells by suppressing epithelial-mesenchymal transition and MMP2, MMP9. PLoS One.

[31] Church MK, Kolkhir P, Metz M (2018). The role and relevance of mast cells in urticaria. Immunol Rev.

[32] Dong H, Diao H, Zhao Y (2019). Overexpression of matrix metalloproteinase-9 in breast cancer cell lines remarkably increases the cell malignancy largely via activation of transforming growth factor beta/SMAD signalling. Cell Prolif.

[33] Ren F, Tang R, Zhang X (2015). Overexpression of MMP Family Members Functions as Prognostic Biomarker for Breast Cancer Patients: A Systematic Review and Meta-Analysis. PLoS One.

[34] Li L, Fan P, Chou H (2019). Herbacetin suppressed MMP9 mediated angiogenesis of malignant melanoma through blocking EGFR-ERK/AKT signaling pathway. Biochimie.

[35] Bakos RM, Bakos L, Edelweiss MI (2007). Immunohistochemical expression of matrix metalloproteinase-2 and -9 in melanocytic nevi is altered by ultraviolet B. Photodermatol Photoimmunol Photomed.

[36] Henry WS, Hendrickson DG, Beca F (2016). LINC00520 is induced by Src, STAT3, and PI3K and plays a functional role in breast cancer. Oncotarget.

[37] Mei XL, Zhong S (2019). Long noncoding RNA LINC00520 prevents the progression of cutaneous squamous cell carcinoma through the inactivation of the PI3K/Akt signaling pathway by downregulating EGFR. Chin Med J (Engl).

[38] Luan W, Ding Y, Yuan H (2020). Long non-coding RNA LINC00520 promotes the proliferation and metastasis of malignant melanoma by inducing the miR-125b-5p/EIF5A2 axis. J Exp Clin Cancer Res.

[39] Tian K, Sun D, Chen M (2020). Long Noncoding RNA X-Inactive Specific Transcript Facilitates Cellular Functions in Melanoma via miR-139-5p/ROCK1 Pathway. Onco Targets Ther.

[40] Duan S, Yu S, Yuan T (2019). Exogenous Let-7a-5p Induces A549 Lung Cancer Cell Death Through BCL2L1-Mediated PI3Kgamma Signaling Pathway. Front Oncol.

[41] Li JP, Liao XH, Xiang Y (2018). Hyperoside and let-7a-5p synergistically inhibits lung cancer cell proliferation via inducing G1/S phase arrest. Gene.

[42] Babapoor S, Wu R, Kozubek J (2017). Identification of microRNAs associated with invasive and aggressive phenotype in cutaneous melanoma by next-generation sequencing. Lab Invest.

