# Supplementary material for: Bioinformatics analysis of lncRNA‑associated ceRNA network in melanoma
Source: J Cancer. 2021 Mar 15;12(10):2921–32. doi: 10.7150/jca.51851 (PMC8040875; doi:10.7150/jca.51851)
Supplement: Supplementary file 1 — Supplementary figures and tables. [file jcav12p2921s1.pdf]

A

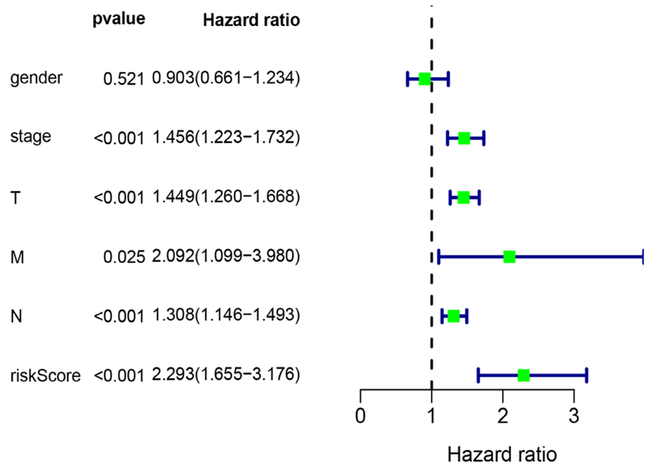

B

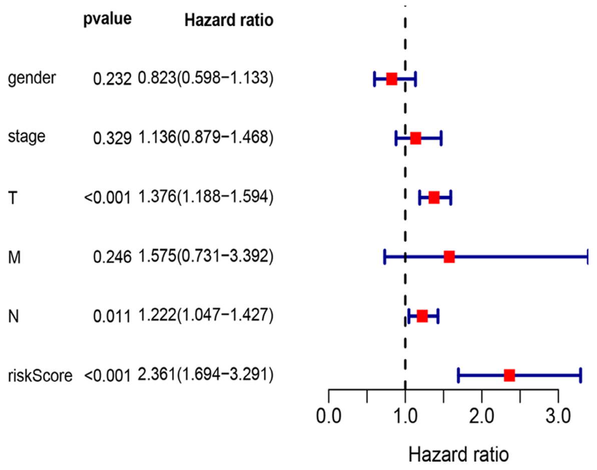

C

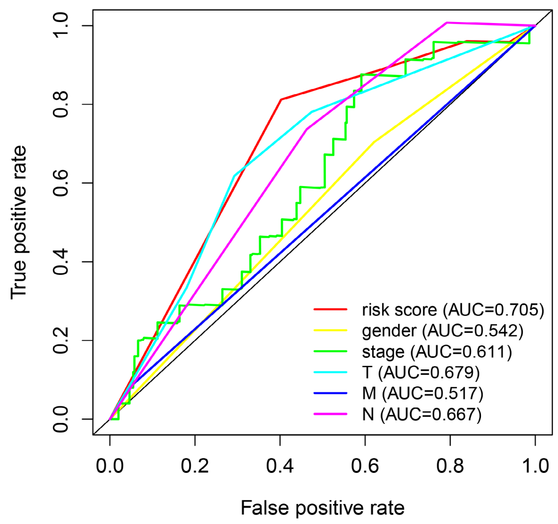

Supplementary Figure 1. Independent prognostic analysis in melanoma. (A) Forest plot of univariate COX analysis. (B) Forest plot of multivariate COX analysis (C) ROC curve and AUC. AUC, area under the curve; ROC, receiver operating characteristics.
